# Research Hotspots and Development Trends on Recycled Construction Materials in Pavement Engineering: A Bibliometric Evaluation

**DOI:** 10.3390/ma14092170

**Published:** 2021-04-23

**Authors:** Yue Xiao, Qiankun Dong, Xiwen Chang, Peiqiang Cui, Gang Liu

**Affiliations:** 1 School of Materials Science and Engineering, Wuhan University of Technology, Wuhan 430070, China; xiaoy@whut.edu.cn (Y.X.); qiankun.dong@whut.edu.cn (Q.D.); liug@whut.edu.cn (G.L.); 2 China Ge Zhou Ba Group Co., Ltd., Wuhan 430079, China; 3 College of Water and Architectural Engineering, Shihezi University, Shihezi 832003, China

**Keywords:** pavement engineering, asphalt pavement, road recycling, recycled materials, bibliometric analysis

## Abstract

Road recycling technology is gradually becoming a research focus in road construction due to natural resource shortages. It is therefore necessary to carry out deep and extensive analysis of the huge amount of publications in the research area of recycling technology in road construction. Based on three databases (Web of Science, Compendex and Scopus) and VOSviewer visualization software, this study conducts a bibliometric analysis of the literature in the field of recycled construction materials in pavement engineering. The global research publications were reviewed to quantitatively identify the literature characteristics. A number of publications, document types, research areas and keywords were used to achieve the general statistics of this reviewed literature. H-index, publication number and citations per publication were used to evaluate the academic contributions by country, institution and journal. The results show that the most productive country and institution for publications are the USA and Chang’an University from China, respectively, followed by China and Wuhan University of Technology. In recent years, researchers have generally paid attention to two main approaches: the application of rubber modified asphalt and the performance enhancement of recycled pavement.

## 1. Introduction

Pavement engineering is an important infrastructure that supports urban development and national progress. At the end of 2020, the total mileage of China’s highways had reached five million kilometers. With the extension of service period, a large number of highways will be in a period of maintenance and reconstruction, which will then result in huge consumption of natural aggregates and serious environmental pollution [1,2,3,4]. Industry solid waste, demolition waste and aged asphalt pavement materials were therefore recycled for pavement engineering due to environmental concerns [5]. For instance, asphalt pavement recycling technology uses a specific process to recycle old asphalt pavement materials to obtain a high performance asphalt mixture [6,7,8,9]. Thereby, the technology can realize the reuse of old pavement materials to achieve waste utilization, as well as reduce production costs [10,11].

Scholars in the field of road construction have conducted all-round research on pavement recycling technology, including the characteristics of waste aggregates, recycling agents, and performance investigation of recycled pavements [12]. Because the incorporation of waste aggregates may have a negative impact on the performance of pavements [13,14,15], some researchers explored the characteristics of recycled pavements to conclude a suitable way to optimize their resulting road performance [16,17,18,19,20], such as mixing amount, mixing temperature, and so on. Wei Minghua [21] gradually determined the gradation and optimal content of each raw material and admixture, and carried out relevant tests to determine the various road performance indicators of the recycled mixture. It provided benefits for the implementation of hot regeneration technology in recycled asphalt mixtures. Li Qiang [22] conducted an in-depth study into the application of high-volume plant-mixed hot-recycled asphalt mixtures. At the same time, recycled asphalt pavement can play a role in reducing environmental pollution and saving natural resources. The research of Wang He [23] illustrated that incorporating an old asphalt mixture in recycled asphalt pavement (RAP) can increase its high-temperature anti-rutting performance. Abedalqader Areej [24] performed performance analysis on old asphalt and recycled aggregates, and used a viscosity-temperature curve method and volume index determination method to determine the optimal construction temperature of recycled asphalt mixtures.

Other researchers have characterized solid waste to clarify its role in reclaimed pavement. Chibuzor O.K. [25] found that the reuse of solid waste can improve the sulfate resistance, heat resistance, temperature resistance, crack resistance, durability, bending resistance, and compressive strength of concrete. Asphalt modification can improve the frost resistance, water permeability, heat resistance, sulfate resistance, flexural tensile strength and durability of pavement [26,27,28,29,30]. Arulrajah [15] compared five types of waste in building materials: recycled concrete aggregate (RCA), broken brick, waste rock (WR), fine recycled glass and RAP. It was found that the water absorption rate of coarse aggregates was lower than that of fine aggregates. In the application of the pavement base, the geotechnical performance of RCA and WR was equivalent to that of stone particle-based materials.

Another key issue of recycled pavement is that the type and dosage of the regenerating agent can affect the performance of recycled asphalt mixture by changing the viscoelastic behavior of the binder. Huang Qibo [31] studied the components of recycled asphalt mixture regenerant. Zhang Zhuang [32] found that the regenerant mixed with a viscosity reducer can effectively improve the low-temperature cracking resistance of aged asphalt, and the high-temperature rutting resistance can also fulfill the standard. Haddadi Sogol Sadat [33] found that the regenerant did not chemically react with the old asphalt. The content of regenerant should be determined according to the performance evaluation of the recycled asphalt mixture.

For the past few years, research on rubber-modified asphalt has gradually become a hot topic. Scholars have studied the existing technologies related to the production, processing and storage of recycled tire rubber modified bitumen (RTR-MB) and its application in pavement engineering. Davide Lo Presti [1] concluded that the wide application of RTR-MB technology in the road paving industry was desirable and a more economical choice could be forwarded. Daryaee Daryoosh [34] found that RTR-MB was a new type of pavement material that could not only improve the service performance, but also alleviate the environmental pollution problem of waste rubber storage.

Worldwide, there is much research on recycled construction materials in pavement engineering, in which the specific directions covered are quite different from each other. The directions include recycled asphalt pavement performance, solid waste utilization, the types and amounts of regenerating agents, rubber-modified asphalt and so on. There are differences and connections between these different directions. In order to have a better understanding of the entire research field, it is necessary to be familiar with the research hotspots and innovative technologies of the current researchers. Bibliometric analysis is one of the effective ways to find the research hotspots and most frequently studied topics in a specific research area.

With the continuous extension of scientific activities, the research findings on recycled asphalt pavement will be more abundant, and the focus of the research will continue to be highlighted in a large amount of literature data. Bibliometrics takes the literature system and bibliometric characteristics as the research object and studies the distribution structure, quantitative relationship, change law and quantitative management of the literature by using mathematics, statistics and other quantitative research methods [35]. It is based on citation analysis, which aims to reveal the development trend of a research field and demonstrate its development. The number of citations and impact factors (IF) of journals are the most common bibliometric evaluation variables [36]. Based on the core database of the Web of Science, this study conducts a visual mapping analysis of the recycling materials in asphalt pavement, aiming to evaluate the bibliometric output of asphalt pavement material recycling technology and its impact [37], so that the research hotspots and development trends of recycled construction materials in pavement engineering can be concluded, according to this bibliometric study.

This research provides data support for both experts and beginners to fully understand the recycled research field by carrying out a bibliometric analysis of papers related to recycled pavement engineering by analyzing its technological development trends and exploring the connections and differences between countries, institutions and journals. Several important evaluated properties and variables that are discussed in this bibliometric evaluation are explained as:

▪ Paper research area: investigated publications are counted by the WoS system, which assigns every journal paper to each research area according to their journal classification.
▪ Paper keyword: counted by occurrences of one keyword in a large number of papers, which can be used to establish the network map of keyword clusters.
▪ H-index: at most H papers have been cited at least H times, which can reflect the level of academic achievement.
▪ Citations per publication (CPP): the ratio of the total number of citations to the number of publications, which can reflect the overall distribution of citations of one country, one institution or one journal.

## 2. Data Resource and Analysis Methodologies

### 2.1. Data Resource

In this study, three database systems, including Web of Science (WoS), Engineering Village (Compendex) and Scopus, were used. For the most comprehensive results, a search across all subscribed resources using a common set of search fields was conducted. The timespan of publications was set from 2000 to 1 February 2020. The key search terms used in this bibliometric were described as TITLE: (Asphalt or bitumen or pavement) and TITLE: (Recycled or recycling or recycle).

These three databases were used to compare the changes in the number of publications over time and the document type of the publications. There were, respectively, 1218, 1508 and 1749 publications in the three database systems, according to the above-mentioned requirements, with different topic, author and other information. The WoS system provides full access to Thomson Reuter’s multidisciplinary databases, including the Science Citation Index Expanded and Social Sciences Citation Index, which includes most important peer-reviewed publications in the research field of road materials and civil engineering. So WoS was then further used for bibliometric analysis with the index of countries, journal titles, institutions, hot topics and most frequently cited papers.

### 2.2. Analysis Methodologies

#### 2.2.1. Visualization Analysis

VOSviewer version 1.6.16 (offered by Leiden University’s Centre for Science and Technology Studies (CWTS), Leiden, The sNetherlands) was used to network analyze the internal relationship of the mass of studied literatures. It can generate a variety of graphs based on bibliometric relationships, such as co-citation graphs of authors and co-occurrence graphs of keywords [38,39]. The map and network of all papers were obtained by the following process: at the beginning, the data of 1218 publications from WoS database were downloaded for VOSviewer analysis. Then, a map was created based on bibliographic data and the file with full data could be analyzed as a network result.

The keyword network can be obtained with Type of analysis: Co-occurrence, unit of analysis: keywords and counting method: full counting. The size of label in the VOSviewer network graph indicates the frequency of the occurrence of this label. The larger the label is, the higher the frequency is. The connection between each label indicates the closeness of the connection between these labels.

#### 2.2.2. Methodologies

With the help of the analysis result function of WoS, Compendex, and Scopus, this study conducts the overall analysis on investigated publications. Firstly, the publication year, document type and research area of all publications were analyzed overall to reveal the trends in the time, type, and application fields of those papers. Publication year and document types were analyzed based on the three database systems, while research area was only analyzed on the WoS system because WoS contains the most important peer-reviewed publications. Secondly, the research hotspot distribution of recycled pavement materials was obtained from all keywords of papers through the data download of endnotes and the analysis of VOSviewer.

Then, single option analysis was performed on the publishing country, affiliated institutions and journals. Three indicators, the number of publications, H-index and citations per publication (CPP), were used to present external factors affecting the publications. Finally, the ten most cited papers were included. Their research content, publication year and affiliated journals were analyzed to explore the research history and future development direction of recycled pavement materials.

## 3. Results Analysis

Based on the three database systems, the annual publications were compared to reveal the differences. Then records obtained from the WoS database were grouped and ranked by means of a detailed analyze results tool. The ranking, record count, percentage, standard research score, and cumulative standard research output score within the obtained records were then plotted and discussed.

### 3.1. General Statistics

#### 3.1.1. Annual Publications

**Figure 1:**
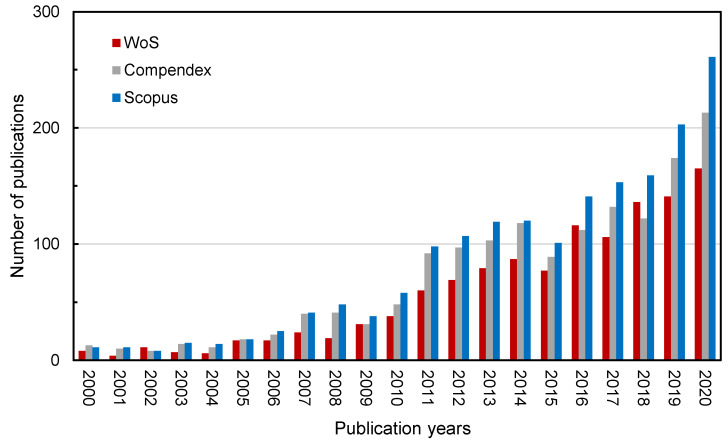
The trends of annual publications in the recycled pavement field.

Figure 1 shows the trends of publications related to the recycled pavement field for the past decades by three database systems. The total number of papers published has increased year by year, and the curve slope in the figure has gradually increased, indicating that the growth rate is accelerating all the time. Papers from WoS database are the least among these three databases because most of WoS database is included in the other two databases, so the publications in this database can also be considered to have higher academic value. As for WoS database, a total of 1218 related papers had been published by the end of 2020. At the same time, the number of annual publications had also shown an increasing trend. Since 2011, the annual number of papers published exceeded 50, in 2016 it exceeded 100, and papers published in 2020 were more than 164. The total number of papers up to 2020 was about 6.7 times that produced up to 2010.

#### 3.1.2. Document Types of Publications

**Figure 2:**
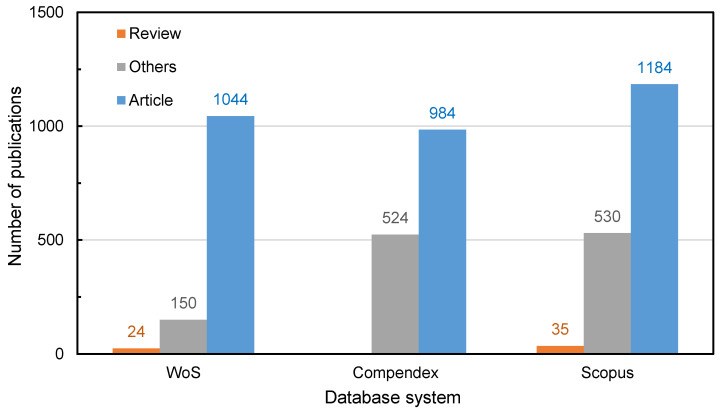
The types of publications in the recycled pavement field.

Figure 2 presents the distribution of publication types, which can be divided into research article, review articles and others. Articles usually accounted for the largest proportion, with 1044, 984 and 1184 publications based on the three databases. These articles mainly focused on a certain research point of recycled materials with experimental exploration and innovation. They introduced a lot of cutting-edge science and technology about recycling. A large number of specific papers were obtained directly when suitable keywords were selected for search and filter. The review article is also a very important part of the publications. Due to the different statistical mechanisms of Compendex, there were no review articles, and the number of articles was relatively small because more articles were classified into “others”, like conference articles and articles in the press. There were 24 and 35 review articles in WoS and Scopus databases, both of which accounted for 2% of the total publications. Although review articles do not report original research data, they are considered as very important publications since they summarize the research, based on which directions for new research as well as supporting the existing theories are proposed. Based on the data of annual publication numbers, an important part of the other types of publications is conference papers, which have shown an increasing trend since 2009. Due to the impact of COVID-19 in the world, the number of conference papers in 2020 decreased. These meeting papers contain abstracts or editorials, excluding the full text. The main content of these publications is basically an exact aspect of the conference theme.

Those review articles from the WoS database can be divided into five types according to their specific directions. Five papers introduced the characteristics of waste aggregates, mainly focusing on their applications and performance. There were nine review papers focusing on the performance evaluation and technical feasibility of recycled pavement (including recycled tire rubber modified asphalt) prepared from various waste materials. Because the deteriorated asphalt material collected from the pavement surface has porous characteristics, it will consequently reduce the road performance of the recycled asphalt. Therefore, research on how to improve the performance of newly designed mixture with recycled materials is a current hot spot and a scientific problem that needs to be solved urgently. There were four papers focusing on cement-related recycling techniques, and another four papers focusing on different recycling technologies. The selection of binders in recycled asphalt concrete and the improvement of recycling methods are also the focus of current research. Cold recycling and warm recycling had one paper each.

#### 3.1.3. Research Areas

**Figure 3:**
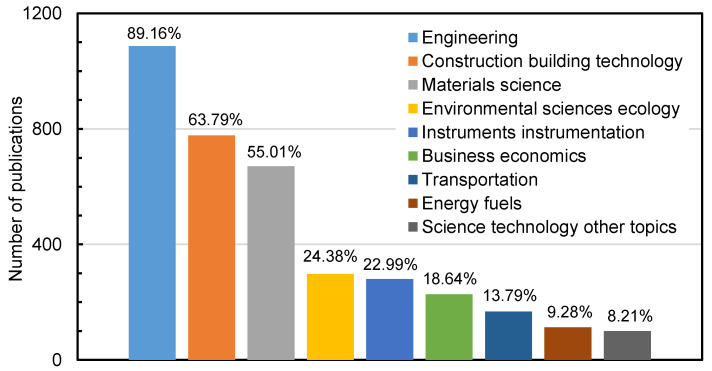
The research areas of publications in the recycled pavement field.

With the help of a category definition from WoS, Figure 3 displays the distribution of the research areas of those investigated publications. The research areas of the investigated publications were counted by the WoS system, which assigned every journal paper to each research area according to their journal classification. Journal papers from multidisciplinary journals were reclassified into specific research areas. The top three research areas were engineering, construction building technology and materials science, which accounted for much more than any other research areas. The papers corresponding to the engineering category were more than 89%, indicating that the final goal of the recycled pavement materials is to solve the engineering problems. Recycling technology has a great significance for road construction, too. The improvement of recycling methods and the research and development of equipment have become the focus of current research on recycled road materials, and construction building technology has become the research direction accounting for 64%. Asphalt material is a traditional pavement material, which needs to realize innovative improvement in order to adapt to contemporary society. There were many papers belonging to materials science, accounting for 55% of the total papers. Similarly, based on the nature of regeneration, many studies also focus on environmental science, transportation and energy fuels.

#### 3.1.4. Keyword Distribution

According to the correlation between keywords, the investigated publications are a network consisting of eight clusters, according to the VOSviewer analysis. Each cluster is a series of keywords obtained by inter-correlating the main keywords of the studied articles. Keywords in the same cluster are more closely related, while the connections between different clusters are lower. The main vocabulary of each cluster is shown in Table 1 by counting the high-frequency vocabulary of each cluster. There is a close relationship between keywords in the same cluster, which can be summarized into the following eight clusters according to their content. The first cluster mainly focuses on the analysis of various performance indicators of recycled concrete aggregate, including compressive strength, resilient modulus, shear strength, flexural strength, permeability, adaptability and mechanical strength. This cluster accounts for the largest proportion of the total keywords, which means that solving road performance is still a hot spot in the current road regeneration problem. The second cluster explores various regenerating agents and modifiers, while the third cluster mainly covers concrete road regeneration materials such as cement, foamed asphalt, and emulsified asphalt. These three clusters of keywords indicate that the current research on recycled pavement is still interested in the design and preparation of recycled asphalt and improvement of various performance indicators of recycled asphalt pavement. The other five clusters include cold recycling mixture design, warm and hot mixture asphalt, environmental impact, service conditions, recycled materials and other related research topics.

**Figure 4:**
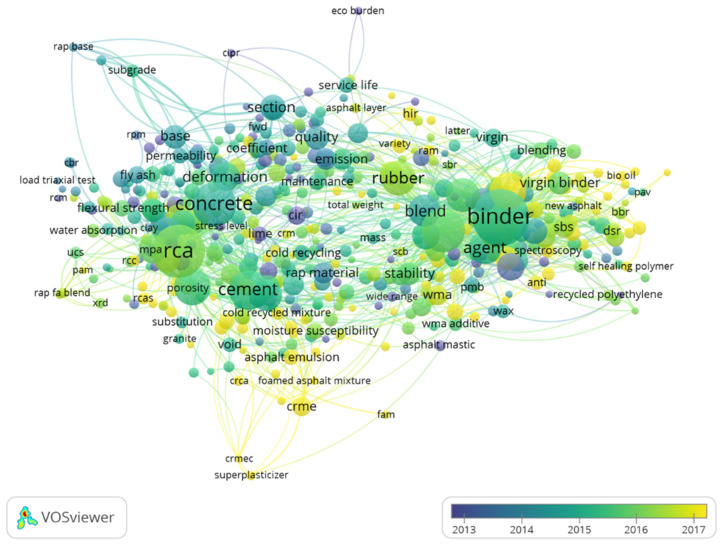
The keyword-year distribution of publications in the recycled pavement field.

Figure 4 shows the keywords of those studied publications with time variation, showing the change of keywords over time. In the figure, keywords were colored in the overlay visualization. If items had scores, the color of a keyword was determined by the score of this keyword, whereby the colors ranged from blue (lowest score) to green to yellow (highest score). From the initial words “eco burden”, it gradually transformed into the development of variously modified asphalts, and then to various comprehensive research on various new asphalt regeneration technologies and environmentally friendly asphalt (low emission). The overall development trend also means that recycled pavement is continuously developing in the direction of more efficiency and emission reduction in order to become more acceptable with the social development.

### 3.2. Statistics of Countries

**Figure 5:**
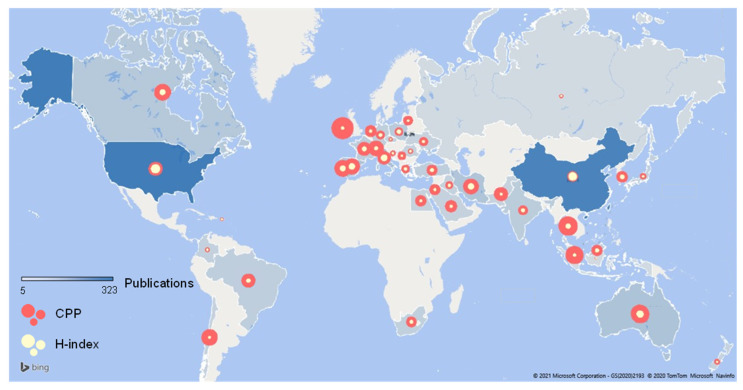
The publication distribution of countries.

The investigated publications were classified and counted by country and the publication distribution map is shown in Figure 5. The largest number of publications was mainly concentrated in Eurasia. North America, due to the two major productive countries, USA and Canada, also had a very strong publication competitiveness. Among them, USA and China ranked as the top two most productive countries for publications with 323 and 297 publications, respectively. Italy (70 papers), Australia (61 papers), Spain (56 papers), Iran (52 papers) and Canada (40 papers) followed closely behind, but the number of publications was much lower than those of the top two countries.

**Figure 6:**
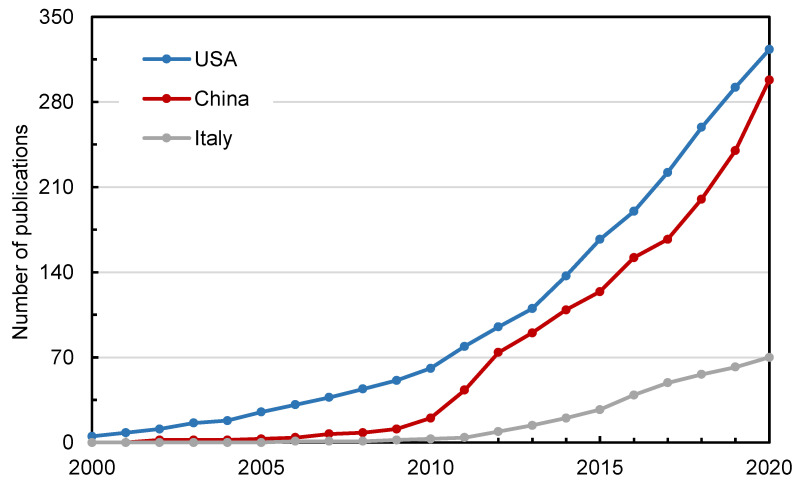
The publication trends of USA, China and Italy from 2000 to 2020.

Figure 6 shows the comparison of the top three countries in terms of publication volume trend. The publications of the USA were accelerating quickly, while the number of papers in China was also continuing to grow with a higher growth rate in 2010 and 2017. The number of publications published in other countries was increasing steadily, indicating that research on recycled pavement materials was still a hot topic.

The H-index can indicate the level of academic achievement. Generally, the higher the H-index, the greater the influence of the paper. For countries, the H-indexes of USA, China and Australia were 34, 28 and 20, respectively, ranking in the top three.

The countries with the highest CPP were Ireland, England and Thailand. This is somewhat different from the data of the H-index. The H-index focuses on the publication of high-level papers, including both the number of publications and the level of papers. CPP can reflect the overall distribution of citations of countries. Especially, the H-index of Ireland and Thailand was not at the forefront even though they had high CPP, due to the small number of publications.

In contrast, in the field of road recycling research, USA, China, Canada, and many European countries such as Italy have strong research capabilities. The scholars may be familiar with the productive countries and regions in this field. At the same time, researchers can learn more about new science and technology through research tracking in these countries.

**Figure 7:**
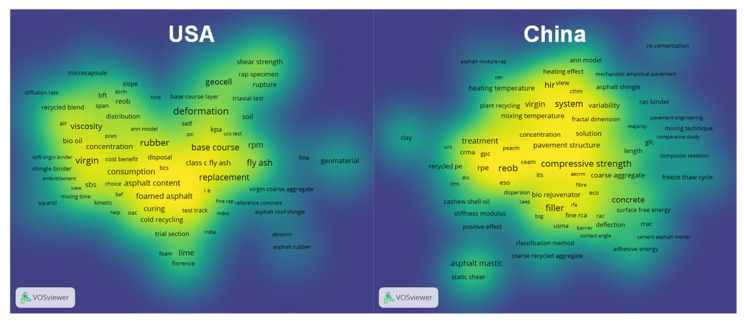
The differences in keyword distribution between the USA and China.

Figure 7 shows the distribution of paper keywords in the two most productive countries for publications, USA and China. The larger the number of keywords in the neighborhood of a point, the closer the color of the point is to yellow. In contrast, the closer the color of the point is to blue, the lower frequency the keywords appear. Both of them paid much more attention to the topic of modified asphalt, such as rubber asphalt and foamed asphalt, and the mechanical properties of recycled pavement, such as compressive strength. It illustrates that these directors are the main topic of current research.

### 3.3. Statistics of Institutions

**Figure 8:**
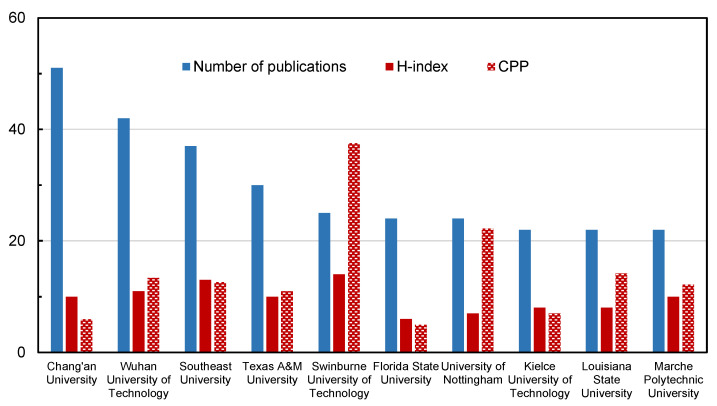
The detailed data of institutions.

The top ten most productive institutions for publications are shown in Figure 8. The ten institutions came from six countries, including three scientific institutions in USA and China, and one each in Australia, England, Poland and Italy. These countries were also at the forefront of productive countries, as Figure 5 shows. It could be considered that these institutions have a major influence on the research field of recycled pavement materials.

The top three productive institutions for publications were all Chinese universities, ranked as Chang’an University (Xi’an 710054, 51 papers), Wuhan University of Technology (Wuhan, 430070, 42 papers) and Southeast University (Nanjing 211189, 37 papers). As prestigious research institutions in the road construction industry, their H-index was also higher, and the influence of the academic papers was greater. Chang [40] analyzed the publication of papers in the field of asphalt research by bibliometric methods, showing the same top four research institutions in the pavement field as this study. It indicates that the top four institutions have world-leading research facilities for the research subject of road engineering, which can therefore ensure a better platform for the corresponding scientific workers.

The H-index gap between the various institutions was not large. The top three institutions with the highest H-index were Swinburne University of Technology (Melbourne, 3122) in Australia, Southeast University and Wuhan University of Technology in China. At the same time, the CPP of Swinburne University of Technology was 37.52, much higher than that of the other institutions, about 2–5 times that of other institutions. It illustrated that every publication from these institutes attracted many more citations than papers from other institutes. Papers from these institutions had a strong guiding significance for future prospective research directions in the research field of recycling techniques in asphalt pavement.

The research situation of the country is based on the publication situation of various research institutions in that country, so the number of publications and the quality of the papers published by institutions are similar to the previous countries research. These data can help scholars in related fields to find scientific research institutions suitable for their own future development. More importantly, a deeper understanding of the research situation of institutions can provide data support for follow-up studies of cutting-edge technology and the research progress of different influential research institutions.

### 3.4. Statistics of Journals

**Figure 9:**
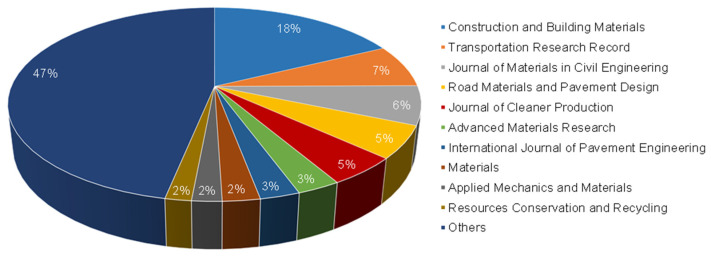
Publication percentages of top productive journals.

Journals publishing these research papers on recycled road materials can provide a submission choice for junior researchers, and can also help researchers to conduct in-depth exploration of papers in this field more quickly. The publication percentages of the top ten productive journals are shown in Figure 9. Among them, Construction and Building Materials ranks No.1 with 216 papers, accounting for 18% of the total publications, which is about 2.5 times that of Transportation Research Record (TRR, No.2). The Journal of Materials in Civil Engineering (JMCE), Road Materials and Pavement Design (RMPD), Journal of Cleaner Production (JLCP), International Journal of Pavement Engineering (IJPE) follow closely, which are all well-known journals in the field of road construction. There are also conference proceedings, including Advanced Materials Research (AMR) and Applied Mechanics and Materials (AMM). The open access journal, Materials, which belongs to the publisher, MDPI, has launched a large number of Special Issues on pavement related topics in recent years, so it attracts many manuscripts.

The influence of the journal in which the paper is published determines the potential influence of the paper. Table 2 shows the H-index and impact factor (IF) of these top productive journals. The data shows that the H-index of CBM, JCP, JMCE and Resources Conservation and Recycling (RCR) is very high, which means that these journals have a large number of high-level publications. Compared with the IF, RCR (IF2020 = 8.086), JCP (IF2020 = 7.246) and CBM (IF2020 = 4.419) have much higher IF than other journals. Although the IFs of JMCE (IF2020 = 2.169) and TRR (IF2020 = 1.029) are lower, this does not affect their position as classic journals on the list for road construction materials, and their publications are of vital importance to the development of road construction.

The influence of publications depends not only on the quality of the paper itself, but also depends on the journal. The current research field uses the IF of journals as a criterion for judging the quality of papers, but there are also many journals with a low IF that have significant influence in the road industry. For scholars, the statistics of journals can not only give important information about suitable paper styles of the different journals, but can also be helpful to follow latest research findings more easily and explore the transfer of research hotspots in time.

### 3.5. Most Frequently Cited Papers

The most frequently cited papers can reveal the hot spots and research trends in this research field, which can also guide beginners to quickly understand the research process and future developments. Table 3 includes the top ten papers with the highest citations on recycled pavement materials, including three review papers and seven research papers.

The top three most cited articles were all review papers, with 305, 304 and 256 citations, respectively. Reviews have a more comprehensive and in-depth analysis of the entire field, which can provide other researchers with research directions and reasonable recommendations. Therefore, review papers are generally cited more frequently. The first review titled: A review of the use of recycled solid waste materials in asphalt pavements [2] mainly summarized the recycling feasibility of four types of solid waste materials: waste glass, steel slag, tires and plastics, including their related technical requirements, standards and documents and the performance of asphalt pavements constructed using such recycled materials. Lo Presti Davide [1] conducted statistical analysis on the existing technologies related to the production, processing and storage of recycled tire rubber modified bitumen and their current applications in road asphalt mixtures. It also clarified the benefits and problems related to the technology and gave suggestions for the widespread use of these technologies. Shu Xiang [4] summarized the latest developments in the use of waste tire rubber in asphalt and Portland cement concrete, and introduced two widely used sustainable methods in the paving industry-RAP and warm mix asphalt.

Research papers are more focused on one specific topic and they can also reflect the current hot spots. Three papers [13,41,42] of these seven articles focused on the performance testing of recycled asphalt mixtures, including fatigue characteristics, the mechanical properties of Portland cement concrete mixed with RAP, and the viscosity of recycled polymer-modified asphalt at different temperatures. There were two papers [43,44] focusing on recycled aggregates, which carried out a comprehensive evaluation of the geotechnical and earth environmental characteristics of different recycled aggregates and explored the changes in resilient modulus and creep values. Zaumanis Martins [45] compared the regeneration effects of five types of rejuvenators and analyzed their rutting resistance, fatigue life and critical cracking temperature. Cao Weidong [46] explored the properties of recycled tire rubber modified asphalt mixtures using a dry process. His research directions included the development of new asphalt materials, the optimization and design of asphalt pavement structure, and the diagnosis and repair of pavement diseases. In the last few years, he has made important contributions to the research of the key technologies and environmental protection measures, like red mud and waste tire rubber powder, and won the second prize of 2016 China Highway Society Science and Technology Progress.

**Table 3:** The basic information of ten most frequently cited papers.

No.	Title	First Author	Year	Source Title	Cited
1	A review of the use of recycled solid waste materials in asphalt pavements	Huang Yue [2]	2007	RCR	305
2	Recycled tire rubber modified bitumens for road asphalt mixtures: a literature review	Lo Presti Davide [1]	2013	CBM	304
3	Recycling of waste tire rubber in asphalt and Portland cement concrete: an overview	Shu Xiang [4]	2014	CBM	256
4	Laboratory evaluation of fatigue characteristics of recycled asphalt mixture	Shu Xiang [41]	2008	CBM	176
5	Geotechnical and geoenvironmental properties of recycled construction and demolition materials in pavement subbase applications	Arulrajah A. [43]	2013	JMCE	165
6	Influence of six rejuvenators on the performance properties of reclaimed asphalt pavement (RAP) binder and 100% recycled asphalt mixtures	Zaumanis Martins [45]	2014	CBM	153
7	Study on properties of recycled tire rubber modified asphalt mixtures using dry process	Cao Weidong [46]	2007	CBM	141
8	Effects of recycled concrete aggregates on properties of asphalt concrete	Paranavithana Sumeda [44]	2006	RCR	140
9	Laboratory investigation of Portland cement concrete containing recycled asphalt pavements	Huang Baoshan [42]	2005	Cement and Concrete Research	138
10	Viscous properties and microstructure of recycled EVA modified bitumen	Garcia-Morales M [13]	2004	Fuel	134

From the perspective of publication time, most of these ten papers were published before 2010. With the continuous advancement of scientific research, the research thinking and research conclusions of this literature are still constantly providing support for subsequent in-depth research. Four papers were published in 2013 and 2014, and the newer papers indicated the continuous expansion of scientific research.

## 4. Conclusions

Based on WoS, Compendex and Scopus databases, there are respectively 1218, 1508 and 1749 publications, from 2000 to 2020, related to the research field of recycled construction materials in pavement engineering. This study analyzed the annual trend of publication numbers and paper types in different databases. Then, a bibliometric analysis of papers in the field of recycled construction materials in pavement engineering was conducted, which can give suggestions for future research on recycled research. According to the discussed statistical analysis, the following conclusions can be drawn.

1. The visualization data analyzed by VOSviewer showed that the keywords of the studied publications could be divided into eight clusters, including performance characterization of RCA, regenerating agents and modifiers, road regeneration materials, environmental impact, emulsifier, ecology burden, subgrade and the variability of RAP. The most focusing topic of this field is the change with time, from the development of modified asphalt to comprehensive research on asphalt regeneration technologies.

2. The investigation of the country of the publications reflected the research stage in this field in different countries. A large number of publications were mainly concentrated in Eurasia. The top three productive countries for publications are USA, China and Italy. The number of publications from the USA and China is much higher than that of other countries. The H-indexes of the USA, China and Australia are ranked in the top three, and are 34, 28 and 20, respectively.

3. The ten most productive institutions for publications come from six countries, including three scientific institutions in the USA and China, and one each in Australia, England, Poland and Italy. The research directions of different research institutions are different with certain differences in the quantity and quality of publications. Learning more about research institutions and their recycled pavement research can provide support for researchers catching up on the latest research findings from classical research institutes with top reputations.

4. Comparing different journals, the data show that CBM, JCP, JMCE and RCR have published many high-level papers. Three of the four journals have a high IF, including RCR (IF2020 = 8.086), JCP (IF2020 = 7.246) and CBM (IF2020 = 4.419). IF is not the only indicator for evaluating the quality of a journal. Although the IF of JMCE and TRR are lower than 2.2, they are also classic journals with vital importance to road construction. With the statistics of journals, it is easier for researchers to gain more information about the different journals and follow the latest research findings and explore the change of research hotspots without delay.

5. The most frequently cited papers indicated that the reuse of solid waste materials, such as waste tire rubber and steel slag, was the hottest direction for research on recycled asphalt pavement. Characterization of recycled asphalt mixtures, recycled aggregates and rejuvenators are the most focused direction trends for the future.

## Figures and Tables

**Table 1:** Eight clusters of keywords from 1218 publications.

Cluster	Keywords	Cluster	Keywords
1. Performance characteristics	RCA Compressive strength Resilient modulus Shear strength Flexural strength Permeability	5. Mixture design	Wheel tracking test Emulsifier Hot Mixture Asphalt Warm Mixture Asphalt
2. Regenerating agents and modifiers	SBS Rubber Blend Rejuvenator Polymer	6. Eco-performance characteristics	Eco burden Fatigue property Recycled hot mix asphalt Water stability
3. Cold recycling	Cement Foamed asphalt Emulsified asphalt Cold recycled mixture Asphalt emission	7. Service conditions	Base, geocell Deformation Subgrade Traffic loading
4. Maintenance aspect	Rehabilitation, maintenance Service life, measurement Asphalt layer thickness Environment impact	8. Recycled materials	Variability Rehabilitation Asphalt Mixture RAP

**Table 2:** Journal statistics of publications in the recycled asphalt field.

Journals	Publications	H-Index	Citations	CPP	IF
Construction and Building Materials	216	40	5554	25.71	4.419
Transportation Research Record	87	16	877	10.08	1.029
Journal of Materials in Civil Engineering	79	19	1272	16.10	2.169
Road Materials and Pavement Design	66	16	670	10.15	2.582
Journal of Cleaner Production	58	21	1097	18.91	7.246
Advanced Materials Research (conference proceeding)	35	2	28	0.80	—
International Journal of Pavement Engineering	32	8	208	6.50	2.646
Materials	30	8	108	3.60	3.057
Applied Mechanics and Materials (conference proceeding)	24	3	25	1.04	—
Resources Conservation and Recycling	21	18	1335	63.57	8.086

## Data Availability

Data supporting reported results can be found in Web of Science.

## References

[1] Presti D.L. (2013). Recycled Tyre Rubber Modified Bitumens for road asphalt mixtures: A literature review. Constr. Build. Mater..

[2] Huang Y., Bird R.N., Heidrich O. (2008). A review of the use of recycled solid waste materials in asphalt pavements. Resour. Conserv. Recycl..

[3] Zou G.L., Sun X.K., Liu X.H., Zhang J.J. (2020). Influence factors on using recycled concrete aggregate in foamed asphalt mixtures based on tensile strength and moisture resistance. Constr. Build. Mater..

[4] Shu X., Huang B.S. (2014). Recycling of waste tire rubber in asphalt and portland cement concrete: An overview. Constr. Build. Mater..

[5] Xue Y.J., Wei X.T., Zhao H., Wang T., Xiao Y. (2020). Interaction of spent FCC catalyst and asphalt binder: Rheological properties, emission of VOCs and immobilization of metals. J. Clean. Prod..

[6] Zhang J.H., Li C., Ding L., Zhao H.G. (2020). Performance evaluation of strengthening recycled coarse aggregate in cement stabilized mixture base layer of pavement. Adv. Civ. Eng..

[7] Ma Y., Polaczyk P., Park H., Jiang X., Hu W., Huang B.S. (2020). Performance evaluation of temperature effect on hot in-place recycling asphalt mixtures. J. Clean. Prod..

[8] Chai L.J., Guo L.P., Chen B., Wang M., Carpinteri A., Scorza D., Vantadori S. (2020). Fracture mechanics-based mixture optimization of ecological high-ductility cementitious composites modified with recycled asphalt concrete. Constr. Build. Mater..

[9] Peter M., Muhammad R.K., Yin P.Z., Bueno M., Poulikakos L. (2020). Incorporation of recycled concrete aggregate (RCA) fractions in semi-dense asphalt (SDA) pavements: Volumetrics, durability and mechanical properties. Constr. Build. Mater..

[10] Sheng Y., Jia H., Lv H., Chen H.X., Zhao X.R., Wang R.Z., Meng J.D. (2020). Study on mesoscopic mechanics of recycled asphalt mixture in the indirect tensile test. Math. Probl. Eng..

[11] Cao Z., Huang X., Yu J., Han X.B., Wang R.Y., Li Y. (2020). Study on all-components regeneration of ultraviolet aged SBS modified asphalt for high-performance recycling. J. Clean. Prod..

[12] Tao G.Y., Xiao Y., Yang L.F., Cui P.D., Kong D.Z., Xue Y.J. (2019). Characteristics of steel slag filler and its influence on rheological properties of asphalt mortar. Constr. Build. Mater..

[13] Garcia-Morales M., Partal P., Navarro F.J. (2004). Viscous properties and microstructure of recycled EVA modified bitumen. Fuel.

[14] Mills-Beale J., You Z. (2010). The mechanical properties of asphalt mixtures with Recycled Concrete Aggregates. Constr. Build. Mater..

[15] Arulrajah A., Disfani M.M., Horpibulsuk S., Suksiripattanapong C., Prongmanee N. (2014). Physical properties and shear strength responses of recycled construction and demolition materials in unbound pavement base/subbase applications. Constr. Build. Mater..

[16] Giani M.I., Dotelli G., Brandini N., Zampori L. (2015). Comparative life cycle assessment of asphalt pavements using reclaimed asphalt, warm mix technology and cold in-place recycling. Resour. Conserv. Recycl..

[17] Thakur J.K., Han J., Pokharel S.K., Parsons R.L. (2012). Performance of geocell-reinforced recycled asphalt pavement (RAP) bases over weak subgrade under cyclic plate loading. Geotext. Geomembr..

[18] Leng Z., Padhan R.K., Sreeram A. (2018). Production of a sustainable paving material through chemical recycling of waste PET into crumb rubber modified asphalt. J. Clean. Prod..

[19] Horpibulsuk S., Hoy M., Witchayaphong P., Rachan R., Arulrajah A. (2017). Recycled asphalt pavement-fly ash geopolymer as a sustainable stabilized pavement material. IOP Conf..

[20] Baghaee M.T., Baaj H. (2016). The use of rejuvenating agents in production of recycled hot mix asphalt: A systematic review. Constr. Build. Mater..

[21] Wei M.H., Wu S.P., Zhu L., Li N., Yang C. (2021). Environmental impact on VOCs emission of a recycled asphalt mixture with a high percentage of RAP. Materials.

[22] Li Q., Sun G.X., Lu Y., Meng Y.P., Luo S., Gao L. (2021). Effects of warm-mix asphalt technologies and modifiers on pavement performance of recycled asphalt binders. J. Clean. Prod..

[23] Wang H., Rath P., Buttlar W.G. (2020). Recycled asphalt shingle modified asphalt mixture design and performance evaluation. J. Traffic Transp. Eng..

[24] Abedalqader A., Shatarat N., Ashteyat A., Katkhuda H. (2020). Influence of temperature on mechanical properties of recycled asphalt pavement aggregate and recycled coarse aggregate concrete. Constr. Build. Mater..

[25] Chibuzor O.K., Duc B.V., Obiekwe U., Charle E., Bunyamin S., Manh N.V., Chijioke I., Talal A., Felix S., Wu W. (2019). Sustainable Soils Re-Engineering. Int. J. Low-Carbon Technol..

[26] Chen Z.W., Wu S.P., Xiao Y., Zeng W.B., Yi M.W., Wan J.M. (2016). Effect of hydration and silicone resin on Basic Oxygen Furnace slag and its asphalt mixture. J. Clean. Prod..

[27] Pan P., Wu S.P., Xiao Y., Liu G. (2015). A review on hydronic asphalt pavement for energy harvesting and snow melting. Renew. Sustain. Energy Rev..

[28] Wang H.N., Ma Z.Y., Chen X., Hasan M.R.M. (2020). Preparation process of bio-oil and bio-asphalt, their performance, and the application of bio-asphalt: A comprehensive review. J. Traffic Transp. Eng..

[29] Ingrassia L.P., Lu X.H., Ferrotti G., Canestrari F. (2020). Chemical, morphological and rheological characterization of bitumen partially replaced with wood bio-oil: Towards more sustainable materials in road pavements. J. Traffic Transp. Eng..

[30] Zalnezhad M., Hesami E. (2020). Effect of steel slag aggregate and bitumen emulsion types on the performance of microsurfacing mixture. J. Traffic Transp. Eng..

[31] Huang Q., Qian Z., Hu J., Zheng D., Chen L.L., Zhang M., Yu J.Z. (2020). Investigation on the properties of aggregate-mastic interfacial transition zones (ITZs) in asphalt mixture containing recycled concrete aggregate. Constr. Build. Mater..

[32] Zhang Z., Han S., Han X., Dong S.H., Yao T.F. (2020). Performance changes of hot recycled asphalt mixture in different layers under coupling of multiple aging factors. Constr. Build. Mater..

[33] Haddadi S.S., Coleri E., Sreedhar S. (2019). Strategies to improve performance of reclaimed asphalt pavement-recycled asphalt shingle mixtures. Int. J. Pavement Eng..

[34] Daryaee D.D., Habibpour M., Gulzar S., Underwood B.S. (2020). Combined effect of waste polymer and rejuvenator on performance properties of reclaimed asphalt binder. Constr. Build. Mater..

[35] Lorusso F., Inchingolo F., Scarano A. (2020). Scientific production in dentistry: The national panorama through a bibliometric study of Italian academies. Biomed Res. Int..

[36] Park K.M., Park B.S., Park S., Yoon D.Y., Bae J.S. (2017). Top-100 cited articles on headache disorders: A bibliometric analysis. Clin. Neurol. Neurosurg..

[37] Simmons P., McElroy T., Allen A.R. (2020). A bibliometric review of artificial extracellular matrices based on tissue engineering technology literature: 1990 through 2019. Materials.

[38] Lorusso F., Noumbissi S., Francesco I., Rapone B., Khater A.G.A., Scarano A. (2020). Scientific trends in clinical research on zirconia dental implants: A bibliometric review. Materials.

[39] Abejon R. (2021). Self-healing asphalt: A systematic bibliometric analysis for identification of hot research topics during the 2003–2018 period. Materials.

[40] Chang X.W., Zhang R.H., Xiao Y., Chen X.Y., Zhang X.S., Liu G. (2020). Mapping of publications on asphalt pavement and bitumen materials: A bibliometric review. Constr. Build. Mater..

[41] Shu X., Huang B.S., Vukosavljevic D. (2008). Laboratory evaluation of fatigue characteristics of recycled asphalt mixture. Constr. Build. Mater..

[42] Huang B.S., Shu X., Li G.Q. (2005). Laboratory investigation of portland cement concrete containing recycled asphalt pavements. Cem. Concr. Res..

[43] Arulrajah A., Piratheepan J., Disfani M.M., Bo M.W. (2013). Geotechnical and geoenvironmental properties of recycled construction and demolition materials in pavement subbase applications. J. Mater. Civ. Eng..

[44] Paranavithana S., Mohajerani A. (2006). Effects of recycled concrete aggregates on properties of asphalt concrete. Resour. Conserv. Recycl..

[45] Zaumanis M., Mallick R.B., Poulikakos L., Frank R. (2014). Influence of six rejuvenators on the performance properties of Reclaimed Asphalt Pavement (RAP) binder and 100% recycled asphalt mixtures. Constr. Build. Mater..

[46] Cao W.D. (2007). Study on properties of recycled tire rubber modified asphalt mixtures using dry process. Constr. Build. Mater..

